# CX3CR1 is a potential biomarker of immune microenvironment and prognosis in epithelial ovarian cancer

**DOI:** 10.1097/MD.0000000000036891

**Published:** 2024-01-19

**Authors:** Danfeng Shao, Honger Zhou, Huaiying Yu, Xiaoqing Zhu

**Affiliations:** a Department of Gynecology, Hangzhou Fuyang First People’s Hospital, Hangzhou, China; b Department of Gynecology, Second Affiliated Hospital, Zhejiang University School of Medicine, Hangzhou, China.

**Keywords:** CX3CR1, immune, ovarian cancer, prognosis

## Abstract

Immunotherapy is less efficient for epithelial ovarian cancer and lacks ideal biomarkers to select the best beneficiaries for immunotherapy. CX3CR1 as chemokine receptor mainly expressed on immune cell membranes, and combined with its unique ligand CX3CL1, mediates tissue chemotaxis and adhesion of immune cells. However, the immune functional and prognostic value of CX3CR1 in epithelial ovarian cancer has not been clarified. A comprehensive retrospective analysis was performed by using the online database to identify the underlying immunological mechanisms and prognostic value of CX3CR1. The Human Protein Atlas, gene expression profiling interactive analysis, and TISIDB (an integrated repository portal for tumor-immune system interactions) database showed that CX3CR1 expressed higher in epithelial ovarian cancer than that in normal ovarian tissue. Four hundred twenty-two cases from Gene Expression Profiling Interactive Analysis and 1656 cases from Kaplan–Meier plotter database showed higher expression of CX3CR1 (above median) was associated with unfavorable overall survival. TIMER, UALCAN, and TISIDB database were applied to validate CX3CR1 negative impact on overall survival. In addition, correlation analysis showed that the expression level of CX3CR1 was positive association with infiltrating levels of B cells (*R* = 0.31, *P* = 3.10e−12), CD8^+^ T cells (*R* = 0.26, *P* = 7.93e−09), CD4^+^ T cells (*R* = 0.11, *P* = 1.41e−02), macrophages (*R* = 0.32, *P* = 4.29e−13), dendritic cells (*R* = 0.27, *P* = 2.98e−09), and neutrophil (*R* = 0.25, *P* = 3.25e−08) in epithelial ovarian cancer. Therefore, CX3CR1 involved in reshaping the immune microenvironment for epithelial ovarian cancer and maybe a potential immunotherapy target and prognostic marker for ovarian cancer.

## 1. Introduction

Ovarian cancer is the most lethal of all gynecological cancers worldwide. In the United States and China, >21,400 and 57,200 women been newly diagnosed with ovarian cancer.^[1,2]^ Due to nonspecific symptoms and the ovary’s location deep within the pelvic cavity, approximately 60% to 70% of ovarian cancer cases are diagnosed at advanced International Federation of Gynecology and Obstetrics stages (III and IV), resulting in delayed diagnoses and treatments. As a result, the 5-year survival rate for stage III and IV cases is 27% and 13%, respectively.^[3,4]^ Ovarian cancer is a heterogeneous tumor with various histological types. Among them, epithelial ovarian cancer accounts for around 90% of all ovarian malignancies.^[5]^ Currently, debulking surgery and chemotherapy containing platinum remain the standard treatment for epithelial ovarian cancer.^[6]^

Recently, numerous studies have suggested that epithelial ovarian cancer is an immunogenic tumor capable of inducing a spontaneous antitumor immune response in the host.^[7]^ Tumor-infiltrating immune cells are present in almost 50% of primary epithelial ovarian cancer tissues, and a high abundance of CD3^+^ or CD8^+^ T-cell infiltration is significantly associated with a better prognosis in epithelial ovarian cancer.^[8]^ Immunotherapy, like immune checkpoints inhibitors (like PD-1/PD-L1 and CTLA-4), have been approved to treat solid cancers and some of them have been studied for epithelial ovarian cancer.^[9]^ Prospective clinical trials including KEYNOTE-028, JAVELIN Ovarian 100, and IMagyn050 study showed that immunotherapy is less efficient for epithelial ovarian cancer and lacks ideal biomarkers to select the best beneficiaries of immunotherapy.^[10–12]^ Hence, it is of great importance to gain a deeper understanding of the role and mechanism of immunological regulation in the microenvironment of epithelial ovarian cancer and search for immune-related genes and proteins.

Chemokine (C-X3-C motif) receptor 1 (CX3CR1) belongs to the G-protein-coupled receptor (GPCR) superfamily, located on chromosome 3p22.2, and CX3C chemokine fractalkine (CX3CL1) is its only ligand chemokine.^[13]^ CX3CR1 as an essential pleiotropic chemokine receptor expressed on human macrophage, T cells, NK cells, and B cells.^[14]^ In physiology, the CX3CR1-CX3CL1 signaling pathway was involved in chemotaxis, activation, and polarization of many cells, as well as organ development and neovascularization. Its deficiency is detrimental to various acute inflammatory responses and exacerbates inflammation.^[15]^ Recently, it has also been found that CX3CR1 was a marker for T-cell differentiation, and CX3CR1 + CD8 + T cells exhibited strong cytotoxicity in antiviral immunity.^[16]^ Studies also have found in breast, prostate, and pancreatic cancer, CX3CR1 is involved in tumor cells spreading and metastasis.^[17]^ However, the immune functional and prognostic value of CX3CR1 in epithelial ovarian cancer has not been clarified.

Numerous online databases, including GEPIA, cBioPortal, TISIDB (an integrated repository portal for tumor-immune system interactions), and TIMER, were retrospectively explored to verify the role of CX3CR1 in epithelial ovarian cancer. The findings showed that CX3CR1 expression was higher in epithelial ovarian cancer than in normal ovarian tissue and other tumors, and closely related to immune microenvironment regulation. Furthermore, high CX3CR1 expression in ovarian cancer was associated with an unfavorable prognosis.

## 2. Materials and methods

### 2.1. Human Protein Atlas

The Human Protein Atlas (HPA) (https://www.proteinatlas.org/) is a free and user-friendly online tool that utilizes various omics technologies and showing the proteins expression in cells, normal tissues, and cancer tissues, respectively.^[18]^ CX3CR1 protein and mRNA expression level among all the tissues and organs were performed in HPA.

### 2.2. Gene expression profiling interactive analysis

Gene expression profiling interactive analysis (GEPIA) (http://gepia2.cancer-pku.cn, version 2) is a user-friendly web database for exploration of gene expression data from the TCGA and GTEx database.^[19]^ In this study, GEPIA was used to CX3CR1 mRNA expression level in cancer and normal tissues, including ovarian cancer. What’s more, survival analysis in Pan-cancer and ovarian cancer was also performed in GEPIA. Overall survival (OS) and disease-free survival (DFS) analyses were calculated by Kaplan–Meier method with a 50% (Median) cutoff for both low and high expression groups. Hazards ratio (HR) and 95% confidence interval were calculated based on Cox PH model.

### 2.3. Sangerbox database

Sangerbox (http://sangerbox.com/) is a comprehensive and user-friendly online analysis tool for bioinformatics analysis and carry out visualization mapping.^[20]^ Sangerbox applied in this study to analyze CX3CR1 mRNA expression data for different cancer and normal samples. The association between CX3CR1 expression and 29 subpopulations of immune cells were also calculated by Sangerbox database.

### 2.4. cBioCancer Genomics Portal

cBioPortal (http://www.cbioportal.org) is an open-access web database, which provides protein abundance, DNA mutations, copy number changes, methylation, and mRNA expression. And the cBioPortal data were collected from TCGA, ICGC, and GEO databases.^[21]^ In addition to gene expression data, this tool can also perform survival analysis. Genetic alterations of CX3CR1 were visually and comparably exhibited by cBioPortal website.

### 2.5. TISIDB

TISIDB (http://cis.hku.hk/TISIDB) is a comprehensive user-friendly online tool explore comprehensive investigation for tumor immunity.^[22]^ CX3CR1 mRNA expression in different stages, grades, and subtypes of ovarian cancer was explored by TISIDB. As well as the relationship between CX3CR1 mRNA expression and immune cells abundance and tumor immune microenvironment factors across multiple cancer types was displayed in the indicated heatmap. Confirmatory analysis of CX3CR1 for overall survival also performed by TISIDB.

### 2.6. Kaplan–Meier plotter

The Kaplan–Meier plotter (http://kmplot.com) analysis is capable to evaluate the correlation between the gene expression and survival for certain types of cancers.^[23]^ Sources for this database was obtained from GEO, EGA, and TCGA. Kaplan–Meier plotter was applied to calculate the relationship between CX3CR1 and survival data (OS and PFS), especially under different clinicopathological features.

### 2.7. Tumor IMmune Estimation Resource database

Tumor IMmune Estimation Resource (TIMER) database (https://cistrome.shinyapps.io/timer/) is a comprehensive online database for analysis of the level of immune cells infiltrating across multitudinous types of cancers.^[24]^ This database not only generate high-quality figures but also hierarchical calculate according to clinical, genomic features and tumor immunological. In this study, the association between CX3CR1 expression level and abundance of immune cells (CD8^+^ T cells, CD4^+^ T cells, B cells, neutrophils, dendritic cells, and macrophages) infiltrating were evaluated using TIMER.

### 2.8. UALCAN database

The UALCAN database (http://ualcan.path.uab.edu) is a publicly available web database containing gene and clinical data for diverse cancers.^[25]^ In this study, UALCAN was visualized to clarify CX3CR1 for overall survival in ovarian cancer.

### 2.9. scTIME portal

Single-cell TIME (scTIME) portal (http://scTIME.sklehabc.com) is a database and a tool for single-cell transcriptomes of tumor immune microenvironment.^[26]^ The scTIME platform collected 49 sets of data from both human and mouse sources, and uniformly labeled cell types for human sources. This platform has built-in common analysis modules, including immune cell type composition and correlation analysis, cell interaction analysis, cell type-specific gene characteristics analysis, and other common modules, all of which can provide convenient retrieval and intuitive analysis results for clinicians or researchers. scTIME portal was used in this study to explore CX3CR1 expressed in different immune cells.

### 2.10. STRING database

STRING online tool (https://string-db.org, version 11.5) is a powerful visualization and customization database to construct protein–protein interactions. In addition to the internal predictions and homologous conversion, STRING depended on numerous resources maintained elsewhere (like COG, Ensembl, PubMed, BioGRID, and KEGG databases).^[27]^ In this study, the STRING portal was applied to evaluate the association between CX3CR1 and functional associated proteins.

### 2.11. Statistical analysis

The study was analyzed using the R software (version 4.2.1). To compare the 2 groups, the Wilcoxon test was employed. The Spearman correlation test was used to evaluate correlations between CX3CR1 expression and related targets. To determine HR, Cox proportional hazards regression models were utilized. Any differences with a *P* value of <.05 were considered statistically significant (*P < .05, **P < .01, ***P < .001).

## 3. Results

**Figure 1.**
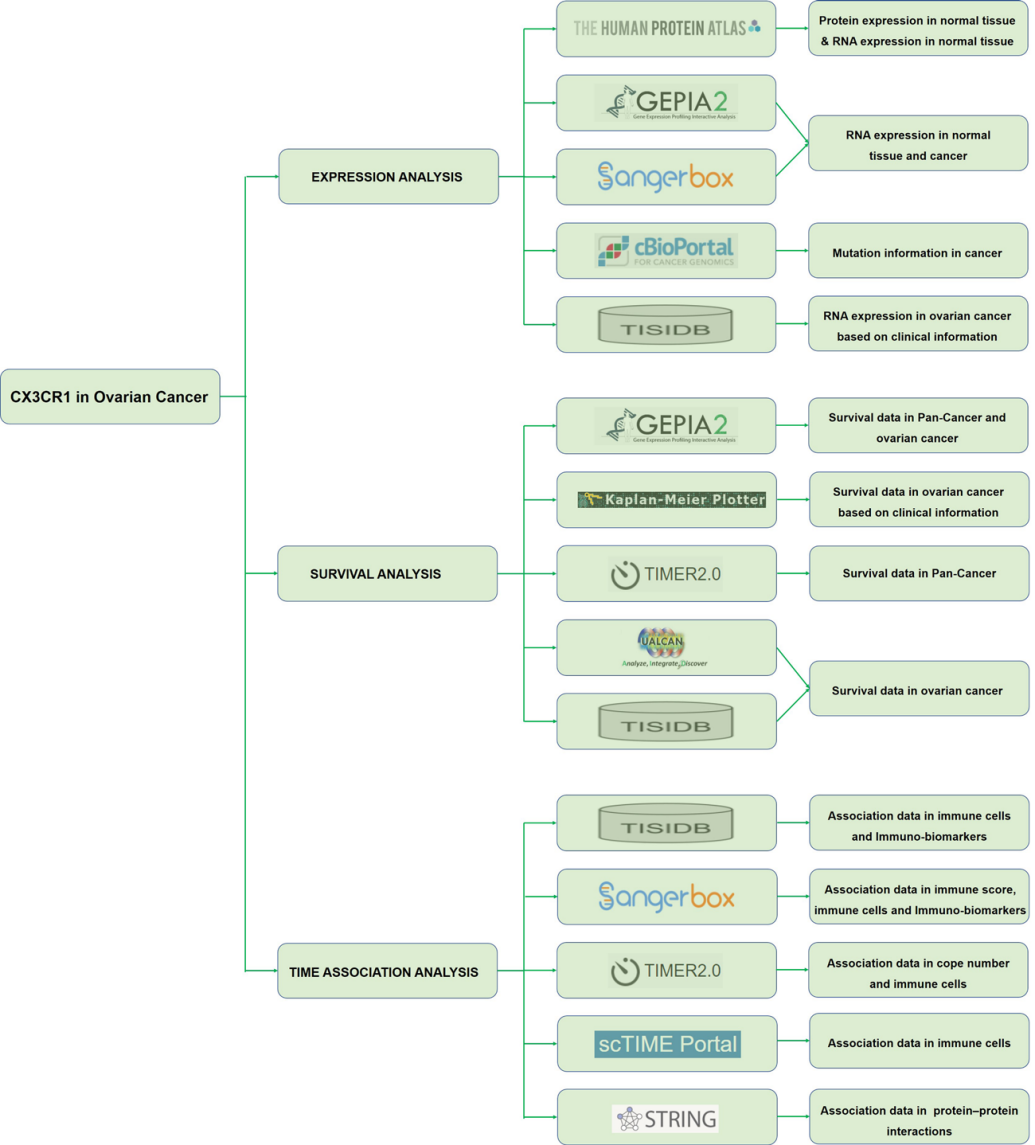
Flowchart of the data preparation and analysis.

The flowchart of the data preparation and analysis showed in Figure 1.

### 3.1. CX3CR1 expression in epithelial ovarian cancer

**Figure 2.**
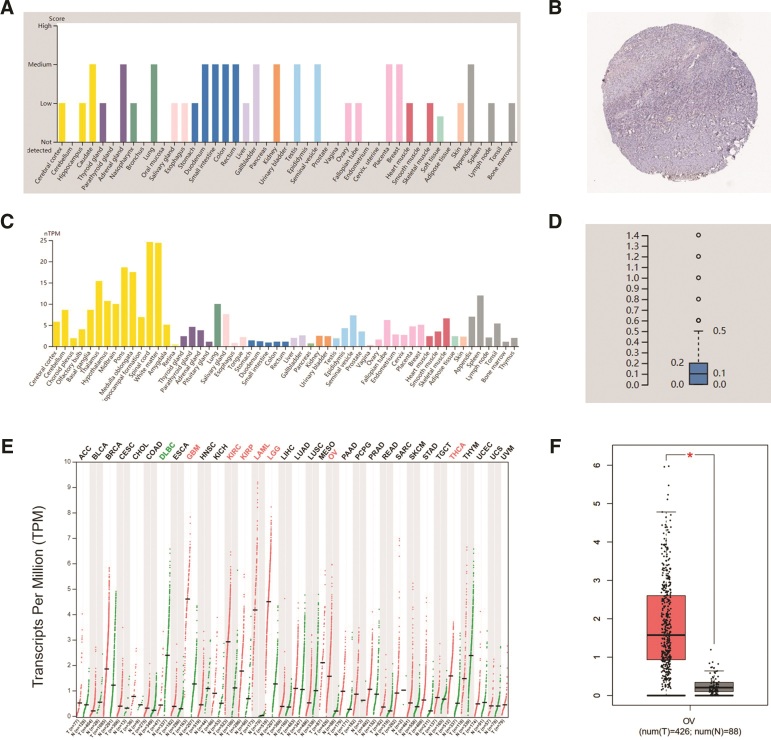
CX3CR1 expression levels in ovarian cancer. (A) CX3CR1 protein expression in normal tissues. (B) CX3CR1 protein expression in normal ovary. (C) CX3CR1 mRNA expression in normal tissues. (D) CX3CR1 mRNA expression in normal ovary. (E) CX3CR1 mRNA expression in Pan-cancer in the GEPIA database. (F) CX3CR1 mRNA expression in epithelial ovarian cancer compared with normal tissues in the GEPIA database. GEPIA = gene expression profiling interactive analysis.

**Figure 3.**
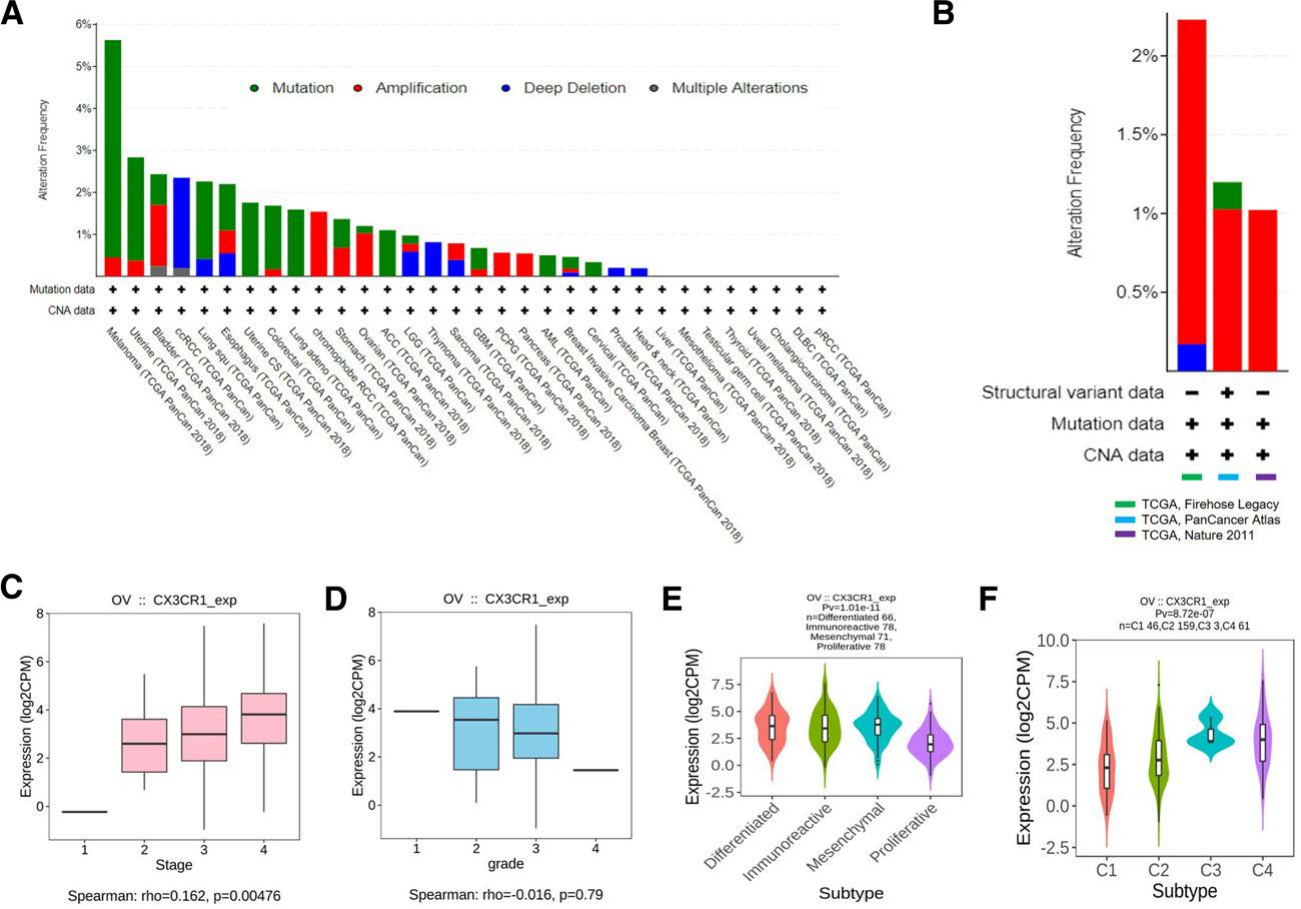
(A) CX3CR1 mutation in Pan-cancer from cBioPortal database. (B) CX3CR1 mutation in ovarian cancer from cBioPortal database. (C) CX3CR1 expression in different stages of epithelial ovarian cancer from GEPIA database. (D) CX3CR1 expression in different grade of epithelial ovarian cancer from GEPIA database. (E) CX3CR1 expression levels in different molecular subtypes of epithelial ovarian cancer from TISIDB database. (F) CX3CR1 expression levels in different immune subtypes of epithelial ovarian cancer from TISIDB database. GEPIA = gene expression profiling interactive analysis.

Data from the HPA database showed that CX3CR1 was low-medium level expression in both protein and mRNA levels in all normal organs and tissues, and was also low in normal ovarian tissues (Fig. 2A–D). However, CX3CR1 expressed significantly increasing in most tumor tissues. Particularly, CX3CR1 expressed higher in epithelial ovarian cancer than that in normal ovarian tissue (Fig. 2E,F). Data from Sangerbox database also confirmed the above results (data not shown). Due to CX3CR1 gene elevated expressed in epithelial ovarian cancer, the mutation map of CX3CR1 gene in epithelial ovarian cancer through the cBioPortal database was observed, including mutation, amplification, deep deletion, and multiple alterations (Fig. 3A). And in epithelial ovarian cancer, amplification was the main change for CX3CR1 (Fig. 3B).

Furthermore, TISIDB database visualized that CX3CR1 was highest expressed in stage IV epithelial ovarian cancer and lowest in stage I (*P* = .00476; Fig. 3C). But in different grade, the expression of CX3CR1 higher in grades I and II (Fig. 3D). Studies found epithelial ovarian cancer can be clustered to mesenchymal, immunoreactive, differentiated, and proliferative molecular subtypes. TISIDB provided the data visualized the proliferative subtype expressed the lowest CX3CR1 among those 4 molecular subtypes (Fig. 3E). In addition, the study from Thorsson et al^[28]^ distinguished C1 to C6, 6 immune subtypes for ovarian cancer, TISIDB database showed CX3XR1 expressed in C1 (wound healing type), C2 (IFN-γ dominant type), C3 (inflammatory type) and C4 (lymphocyte depleted type) subtypes, and highest expressed in C3 (inflammatory) type of epithelial ovarian cancer (Fig. 3F).

### 3.2. CX3CR1 prognostic value in epithelial ovarian cancer

**Figure 4.**
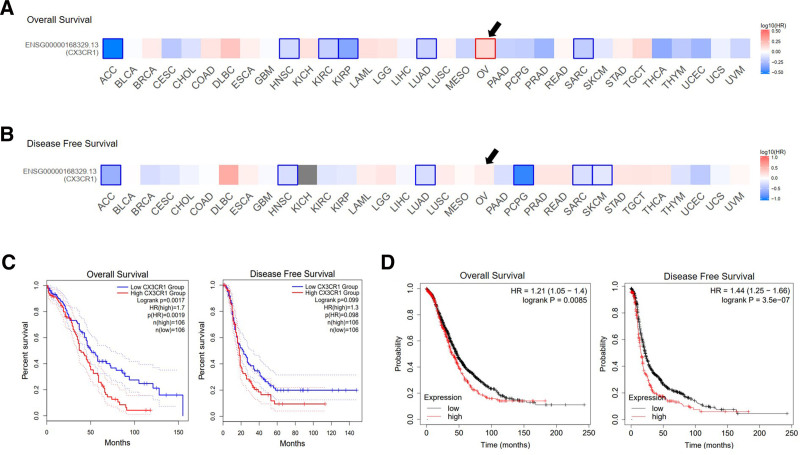
The prognosis value of CX3CR1 in ovarian cancer. (A) CX3CR1 expression and ovarian cancer overall survival among Pan-cancer. (B) CX3CR1 expression and ovarian cancer disease-free survival among Pan-cancer. (C) Kaplan–Meier survival curves comparing the high and low expression of CX3CR1 in epithelial ovarian cancer in the GEPIA. (D) Kaplan–Meier survival curves comparing the high and low expression of CX3CR1 in epithelial ovarian cancer in the Kaplan–Meier plotter databases. GEPIA = gene expression profiling interactive analysis.

The correlation of CX3CR1 with Pan-cancer survival was calculated in GEPIA database, the OS and DFS analysis including 33 cancer types (Fig. 4A,B). Totally, 422 epithelial ovarian cancer cases included, results showed higher expression of CX3CR1 (above median) was associated with a significant shorter OS (HR = 1.4, *P* = .012), but no different for DFS (HR = 1, *P* = .920; Fig. 4C).

The potential prognostic value of CX3CR1 for epithelial ovarian cancer was confirmed in Kaplan–Meier plotter database. Ultimately, 1656 cases included for OS and 1435 patients for progression-free survival (PFS) analysis. The expression of CX3CR1 gene strongly predict unfavorable OS (HR = 1.21 [1.05–1.40], *P* = .0085) and PFS (HR = 1.44 [1.25–1.66], *P* = 3.5e−07; Fig. 4D; Table 1).

**Table 1:** Correlation of CX3CR1 mRNA expression and clinical prognosis in epithelial ovarian cancer with different clinicopathological factors by Kaplan–Meier plotter.

		N	HR, 95%CI	*P*
**Overall survival**				
Total		1656	1.21, 1.05–1.40	.0085
Histology	Endometrioid	37	3.40, 0.57–20.36	.1544
	Serous	1207	1.29, 1.09–1.52	.0029
Stage	I	74	0.06, 0.01–0.45	.0002
	II	61	2.60, 0.81–8.39	.0979
	III	1044	1.28, 1.07–1.54	.0058
	IV	176	1.38, 0.93–2.05	.1080
Grade	Low	56	0.67, 0.22–2.07	.4857
	High	1339	1.28, 1.09–1.50	.0027
P53	Mutated	506	1.31, 1.02–1.68	.0367
	Wild type	94	1.76, 1.01–3.05	.0420
Debulk	Optimal	801	1.42, 1.14–1.78	.0020
	Suboptimal	536	1.34, 1.07–1.69	.0121
**Disease-free survival**				
Total		1435	1.44, 1.25–1.66	3.5e−7
Histology	Endometrioid	51	1.95, 0.69–5.47	.1980
	Serous	1104	1.38, 1.18–1.61	5.2e−5
Stage	I	96	0.18, 0.02–1.41	.0670
	II	67	1.37, 0.65–2.90	.4022
	III	919	1.46, 1.24–1.73	7.6e−6
	IV	162	1.21, 0.81–1.82	.3551
Grade	Low	37	2.73, 0.75–10	.1134
	High	1093	1.42, 1.21–1.66	51.3e−5
P53	Mutated	483	1.43, 1.13–1.81	.0024
	Wild type	84	1.65, 0.92–2.96	.0886
Debulk	Optimal	696	1.60, 1.30–1.98	1.1e−5
	Suboptimal	459	1.52, 1.2–1.93	.0005

CI = confidence intervals, HR = hazard ratios.

The detailed relationship between CX3CR1 and epithelial ovarian cancer survival prognosis under different clinicopathological characteristics was analyzed by using Kaplan–Meier plotter database (Table 1). TIMER, UALCAN, and TISIDB database were applied to validate CX3CR1 negative impact on overall survival (data not shown).

### 3.3. CX3CR1 and immune cells infiltration in epithelial ovarian cancer

**Figure 5.**
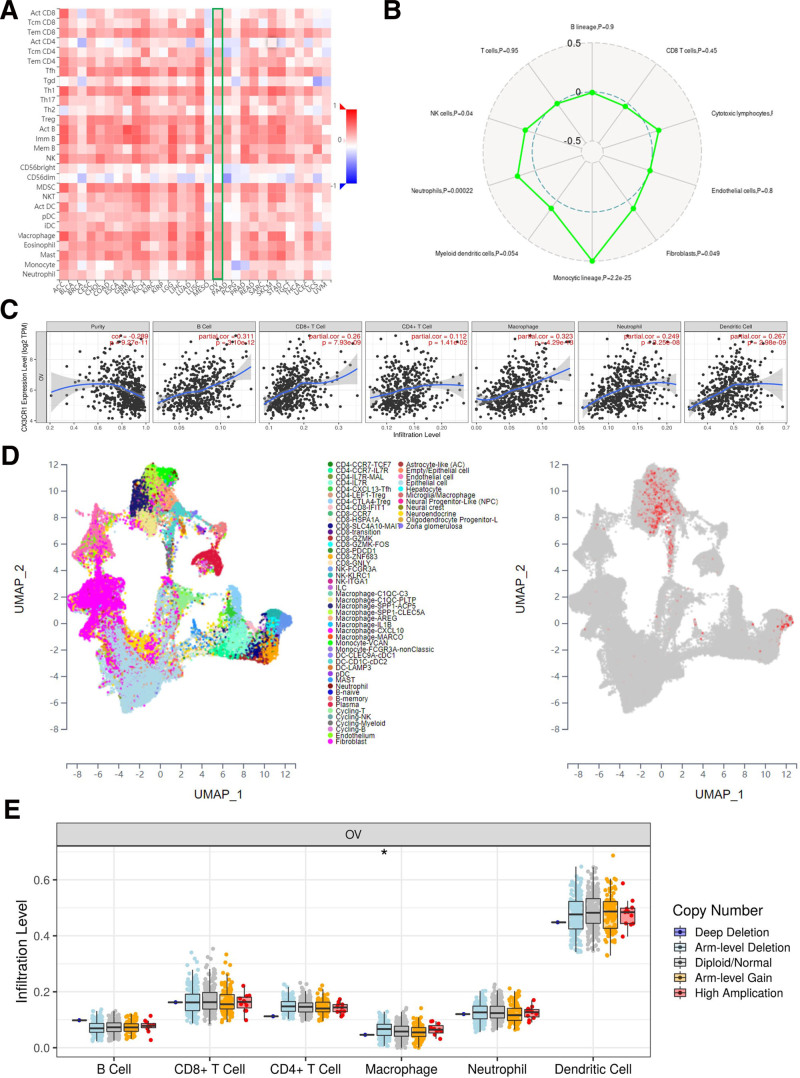
CX3CR1 in immune microenvironment of ovarian cancer. (A) The correction between CX3CR1 and immune cells among Pan-cancer. (B) The correction between CX3CR1 and immune cells in ovarian cancer. (C) The correction between CX3CR1 and 6 immune cells in ovarian cancer from TIMER. (D) CX3CR1 expression among different immune cells in ovarian cancer from scTIME portal. (E) CX3CR1 mutation among immune cells in ovarian cancer. TIMER = Tumor IMmune Estimation Resource database.

The TIMER database was applied to assess the correction between the expression of CX3CR1 gene and lymphocytes abundance in epithelial ovarian cancer. Correlation analysis showed that the expression level of CX3CR1 was positive association with infiltrating levels of B cells (*R* = 0.31, *P* = 3.10e−12), CD8^+^ T cells (*R* = 0.26, *P* = 7.93e−09), CD4^+^ T cells (*R* = 0.11, *P* = 1.41e−02), macrophages (*R* = 0.32, *P* = 4.29e−13), dendritic cells (*R* = 0.27, *P* = 2.98e−09), and neutrophil (*R* = 0.25, *P* = 3.25e−08) in epithelial ovarian cancer (Fig. 5A). Although, CX3CR1 was not correlation with tumor purity (*r* = −0.29, *P* = 9.27e−11). The TISIDB database also verified that CX3CR1 gene expression strongly concerned in lymphocytes infiltration for epithelial ovarian cancer, especially macrophages (*R* = 0.46), MDSC (*R* = 0.47), and activated B cells (*R* = 0.66; Fig. 5B). Interestingly, Sangerbox database also validated the relationship between CX3CR1 with monocytic lineage cells (macrophages) was stronger than other immune cells (Fig. 5C). Data from scTIME portal also shown that CX3CR1 mostly expressed on macrophages (Fig. 5D). Furthermore, TIMER database visualized various forms of CX3CR1 copy number and immune cells immersion in epithelial ovarian cancer, showed that CX3CR1 arm-level depletion was associated with the infiltration of macrophages (Fig. 5E).

### 3.4. CX3CR1 and immune biomarkers in epithelial ovarian cancer

**Figure 6.**
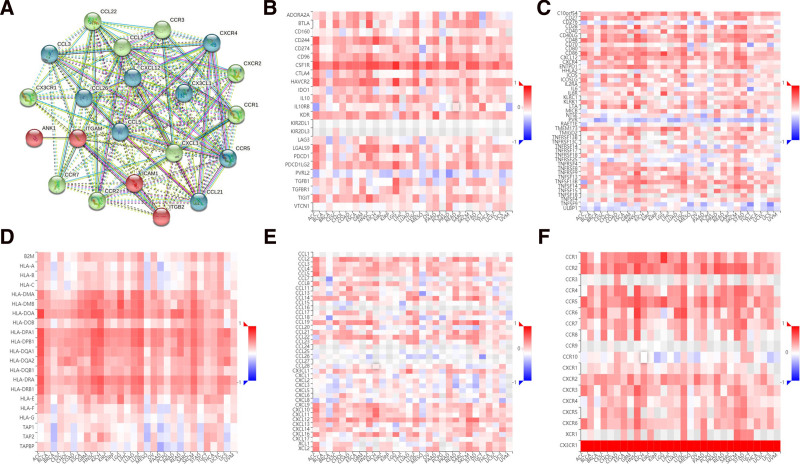
The correction between CX3CR1 and immune biomarkers. (A) PPI network between CX3CR1 and other proteins. (B) Heat map shows the correction between CX3CR1 and immunoinhibitors. (C) Heat map shows the correction between CX3CR1 and immunostimulators. (D) Heat map shows the correction between CX3CR1 and MHC molecules. (E) Heat map shows the correction between CX3CR1 and chemokines. (F) Heat map shows the correction between CX3CR1 and chemokine receptors. MHC = Major Histocompatibility Complex, PPI = protein–protein interaction.

Protein–protein interaction network analysis showed that CX3CR1 correlated with most chemokines and receptors (Fig. 6A). The TISIDB database assessed that CX3CR1 gene expression was significantly associated with immunoinhibitors in epithelial ovarian cancer, including HAVCR2 (*R* = 0.51, *P* < 2.2e−16) and CSF1R (*R* = 0.66, *P* < 2.2e−16; Fig. 6B). CX3CR1 was dim correlated with immunostimulators in epithelial ovarian cancer, such as CD86 (*R* = 0.49, *P* < 2.2e−16) was the most closely related to CX3CR1 (Fig. 6C). Major Histocompatibility Complex molecule also close to CX3XR1 expression in epithelial ovarian cancer, like HLA-DMA (*R* = 0.49, *P* < 2.2e−16) and HLA-DPA1 (*R* = 0.473, *P* < 2.2e−16; Fig. 6D). The association between CX3CR1 gene expression with chemokines was performed in TISIDB database. For instance, CX3CR1 was significantly correlated with CCL2/3 and (*R* = 0.34, *P* < 1.03e−09) and CX3CL1 (*R* = 0.18, *P* < .0015) in epithelial ovarian cancer (Fig. 6E). The expression of CX3CR1 was also significantly related to chemokine receptors, including CXCR2 (*R* = 0.54, *P* < 2.2e−16) and CCR5 (*R* = 0.43, *P* < 2.2e−16) in epithelial ovarian cancer (Fig. 6F).

**Figure 7.**
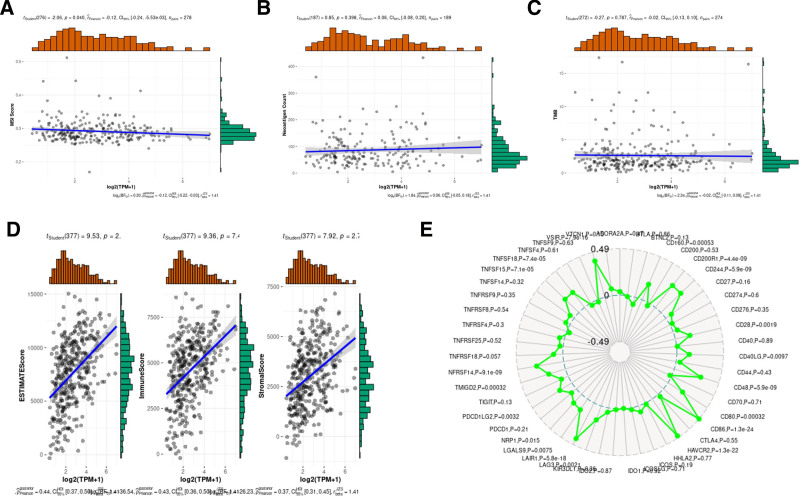
The correction between CX3CR1 and ImmuneScores or StromalScores. (A) Correction between CX3CR1 and MSI scores. (B) Correction between CX3CR1 and neoantigen counts. (C) Correction between CX3CR1 and TMB. (D) Correction between CX3CR1 and ESTIMATEScore, ImmuneScore, and StromalScore. (E) Correction between CX3CR1 and immune points. MSI = microsatellite instability, TMB = tumor mutational burden.

Correlation analysis results also suggested CX3CR1 significantly associated with ESTIMATEScore (*R* = 0.44, *P* < 2.01e−19), ImmuneScore (*R* = 0.43, *P* < 7.43e−19), and StromalScore (*R* = 0.38, *P* < 2.77e−14; Fig. 7D). Tumor mutational burden, microsatellite instability (MSI), and Neoantigen score were genomic biomarkers used to predict the clinical benefit of immune checkpoint inhibitors therapy (Fig. 7A–C). The relationship between CX3CR1 and genomic biomarkers in epithelial ovarian cancer has been calculated, but there was no positive correlation (Fig. 7D,E).

## 4. Discussion

CX3CR1 was a chemokine receptor mainly expressed on immune cell membranes, and combined with its unique ligand CX3CL1, mediates tissue chemotaxis and adhesion of immune cells.^[29]^ Studies found in tumor, CX3CR1 expressed by tumor associated macrophage and tumor cells.^[30]^ However, the real role of CX3CR1 in the epithelial ovarian cancer was still controversial. This study implied that CX3CR1 expression level associated with cancerous prognosis. Higher level of CX3CR1 expression predicted better prognosis of epithelial ovarian cancer and early stage. Besides, this study also showed that immune cells and multitudinous immunomodulators in epithelial ovarian cancer were correlated with expression level of CX3CR1. Therefore, this comprehensive and in-depth study provided new insights into the potential immunomodulatory role of CX3CR1 in the epithelial ovarian cancer microenvironment and suggested it as a biomarker for cancer prognosis.

The expression level of CX3CR1 gene and protein in normal ovaries and epithelial ovarian cancer by applying available web databases in HPA, GEPIA2, Sangerbox, and TISIDB, and CX3CR1 mutation data were analysis in cBioPortal. The expression of the CX3CR1 in epithelial ovarian cancer was higher than that in normal ovaries. Notoriously, epithelial ovarian cancer is a group of heterogeneous malignant tumors and identified into 4 molecular subtypes.^[31]^ The TISIDB database visualized that CX3CR1 expression level strongest associated to immune-related subtypes and lowest expressed in proliferative types. Among the distinct immune subtypes (C1–C6) of epithelial ovarian cancer, C3 (inflammatory type) expressed the highest level of CX3CR1 than other immune subtypes. Another breakthrough point was that CX3XR1 closely associated to the immune cells infiltration and various immune-related biomarkers in epithelial ovarian cancer. Those various online databases reflected CX3CR1 was strongly associated with immunological properties in the epithelial ovarian cancer niche. Furthermore, the CX3CR1 expression level correlation with prognostic value in ovarian cancer by analyzing the data from GEPIA and Kaplan–Meier plotter databases. The increased CX3CR1 expression related to favorable prognosis of epithelial ovarian cancer, which robustly suggested that CX3CR1 can be applied as a promising prognostic biomarker for epithelial ovarian cancer.

## 5. Conclusion

CX3CR1 is a chemokine receptor that plays a role in reshaping the immune microenvironment of epithelial ovarian cancer, and CX3CR1 maybe a potential immunotherapy target and prognostic marker for ovarian cancer, which were worthy for further and deeply study.

## Author contributions

**Investigation:** Danfeng Shao.

**Methodology:** Danfeng Shao, Huaiying Yu, Xiaoqing Zhu.

**Project administration:** Danfeng Shao.

**Software:** Honger Zhou.

**Writing – review & editing:** Danfeng Shao.

**Writing – original draft:** Honger Zhou, Huaiying Yu.
